# Patent Ductus Arteriosus and Bronchopulmonary Dysplasia–Associated Pulmonary Hypertension: A Bayesian Meta-Analysis

**DOI:** 10.1001/jamanetworkopen.2023.45299

**Published:** 2023-11-28

**Authors:** Eduardo Villamor, Elke van Westering-Kroon, Gema E. Gonzalez-Luis, František Bartoš, Steven H. Abman, Maurice J. Huizing

**Affiliations:** 1 Division of Neonatology, Department of Pediatrics, MosaKids Children’s Hospital, Maastricht University Medical Center (MUMC+), School for Oncology and Reproduction (GROW), Maastricht University, Maastricht, The Netherlands; 2 Department of Pediatrics, Hospital Universitario Materno-Infantil de Canarias, Las Palmas de Gran Canaria, Spain; 3 Department of Psychology, University of Amsterdam, Amsterdam, The Netherlands; 4 Pediatric Heart Lung Center, Department of Pediatrics, University of Colorado Anschutz School of Medicine and Children’s Hospital Colorado, Aurora

## Abstract

**Importance:**

Bronchopulmonary dysplasia (BPD) is often associated with pulmonary vascular disease and secondary pulmonary hypertension (PH). The pathogenesis of BPD-associated PH (BPD-PH) is complex and involves prenatal and postnatal factors that disrupt pulmonary vascular development, and patent ductus arteriosus (PDA) is a factor potentially associated with risk of BPD-PH that has been identified in very recent studies.

**Objective:**

To explore the association of PDA with BPD-PH using a bayesian model-averaged (BMA) meta-analysis of studies.

**Data Sources:**

PubMed and Embase were searched up to April 2023. Key search terms included *BPD* and *PH.*

**Study Selection:**

Studies examining infants with gestational age 32 weeks or less and reporting data on PDA and risk of BPD-PH.

**Data Extraction and Synthesis:**

This study followed the Preferred Reporting Items for Systematic Reviews and Meta-Analyses and the Meta-Analysis of Observational Studies in Epidemiology reporting guidelines. Two independent reviewers extracted data, with a third reviewer checking for accuracy and completeness. Data pooling and effect size calculations were performed by BMA.

**Main Outcomes and Measures:**

The primary outcome was BPD-PH. BMA was used to calculate Bayes factors (BFs). The BF_10_ is the ratio of the probability of the data under the alternative hypothesis (H_1_, association of PDA with BPD-HP) over the probability of the data under the null hypothesis (H_0_).

**Results:**

A total of 32 studies (8513 infants) were included. BMA showed that the evidence in favor of H_1_ was weak for any PDA (BF_10_ = 2.90; 10 studies), moderate for hemodynamically significant PDA (BF_10_ = 3.77; 3 studies), and extreme for surgically ligated or catheter-occluded PDA (BF_10_ = 294.9; 16 studies). In contrast, the evidence in favor of H_0_ was weak for medically treated PDA (BF_10_ = 0.55; 6 studies). In addition, BMA found strong evidence in favor of H_1_ when prolonged exposure to PDA was analyzed as a dichotomous variable (BF_10_ = 11.80; 6 studies) and extreme evidence (BF_10_ = 113.60; 3 studies) when PDA exposure time was analyzed as a continuous variable.

**Conclusions and Relevance:**

In this bayesian meta-analysis, the data suggest that prolonged exposure to PDA might be associated with increased risk of pulmonary vascular disease in extremely preterm infants. This highlights the need to monitor for PH in high-risk preterm infants with prolonged exposure to PDA and to incorporate PH risk into clinical decisions regarding PDA management.

## Key Points

**Question:**

Is patent ductus arteriosus (PDA) associated with a higher risk of developing bronchopulmonary dysplasia–associated pulmonary hypertension (BPD-PH) among very and extremely preterm infants?

**Findings:**

In this meta-analysis of 32 studies with 8513 infants, the bayesian model-averaged meta-analysis found associations of BPD-PH with both PDA requiring surgery and prolonged PDA.

**Meaning:**

These findings suggest that sustained patency of a hemodynamically significant ductal shunt may be a key contributor to pulmonary vascular disease in very and extremely preterm infants and highlight the need to monitor for PH in high-risk preterm infants and to incorporate PH risk into clinical decisions regarding PDA management.

## Teaser

This bayesian meta-analysis examines the association of patent ductus arteriosus with bronchopulmonary dysplasia–associated pulmonary hypertension in extremely preterm infants.

## Introduction

Bronchopulmonary dysplasia (BPD) is one of the most common complications of very and extremely preterm birth (ie, gestational age <32 weeks) and is an important contributor to pulmonary and nonpulmonary morbidity and mortality.^1^ BPD is often associated with pulmonary vascular disease and secondary pulmonary hypertension (PH). BPD-associated pulmonary hypertension (BPD-PH) is characterized by arrested vascular growth and abnormal remodeling of the pulmonary vasculature, resulting in high vascular tone and abnormal reactivity, which may lead to increased pulmonary vascular resistance and right heart failure.^2,3^ The pathogenesis of BPD-PH is multifactorial and involves maternal, placental, fetal, and postnatal factors that disrupt fetal and neonatal growth and development of the pulmonary vascular bed.^3,4^ BPD-PH is estimated to be present in 25% of infants with moderate-to-severe BPD, and its presence is associated with worse respiratory outcomes and higher hospital readmission and mortality rates.^1,3,4^

Although there is no consensus on the diagnostic criteria for BPD-PH, a growing number of observational studies have been published comparing infants with PH, as assessed by echocardiography, with infants having BPD without PH. This has allowed the identification of several prenatal and postnatal factors associated with BPD-PH.^5,6,7^ Among these factors are lower gestational age (GA), being small for GA, hypertensive disorders of pregnancy, oligohydramnios, sepsis, higher mechanical ventilation requirements, or BPD severity.^5,6,7^

Preclinical studies^8,9^ have shown that hemodynamic stress impairs pulmonary vascular function, structure, and growth in animal models of perinatal PH, but the impact of a patent ductus arteriosus (PDA) on late clinical outcomes after preterm birth remains uncertain. The presence of or prolonged exposure to a hemodynamically significant PDA (hsPDA) has been suggested as a potential risk factor for developing BPD-PH in some recent cohorts.^10,11^ However, the potential contribution of PDA or its association with the risk of sustained or late PH in infants with established BPD has not been analyzed in the various meta-analyses published to date.^5,6,7^

Our objective for the current study was to conduct a systematic review and meta-analysis of the association of PDA with BPD-PH. Instead of the more commonly used frequentist statistics, we used a bayesian approach for the meta-analysis. In contrast to frequentist null hypothesis (H_0_) significance testing, which focuses exclusively on H_0_, Bayesian hypothesis testing aims to quantify the relative plausibility of the alternative hypothesis (H_1_) and H_0_. Quantification of evidence on a continuous scale allows for more nuanced conclusions than do all-or-none (significant vs nonsignificant) conclusions.^12,13,14^ The bayesian approach may provide a wider, and arguably more informative, set of interpretations than that typically provided by a frequentist analysis.^12,13,14^

## Methods

The method for this study was based on our recently published experience on performing meta-analyses to study the associations of antenatal and perinatal exposures with outcomes of prematurity.^6,15,16,17,18^ Because this meta-analysis did not involve animal subjects or personally identifiable information on human participants, ethics review board approval and patient consent were not required, in accordance with Dutch law regarding human medical scientific research, which is enforced by the Central Committee on Research Involving Human Subjects. The study was performed and reported according to Preferred Reporting Items for Systematic Reviews and Meta-analyses (PRISMA) and Meta-analysis of Observational Studies in Epidemiology (MOOSE) reporting guidelines. The population, exposure, comparison, and outcome question was, do very and extremely preterm infants (population) exposed to PDA (exposure) have a higher risk of developing BPD-PH (outcome) than preterm infants with no history of exposure (comparison)?

### Sources and Search Strategy

A comprehensive literature search was undertaken using the PubMed, EMBASE, and Web of Science databases. The literature search was updated up to February 2023. The search strategy is detailed in the eAppendix in Supplement 1. Studies were included if they examined very and extremely preterm (GA <32 weeks) or very low birth weight (<1500 g) infants and reported primary data that could be used to measure the association of exposure to PDA with the development of BPD-PH.

### Data Extraction, Definitions, and Quality Assessment

Two reviewers (E.V. and E.v.W.-K.) extracted data from relevant studies, and another reviewer (M.J.H.) checked data extraction for accuracy and completeness. Discrepancies were resolved by consulting the primary report. The outcome considered in meta-analysis was BPD-PH, defined by any echocardiographic criteria as long as the evaluation was performed at a postnatal age greater than 4 weeks. The exposure to PDA was divided into 6 groups on the basis of the categories provided in the different studies: (1) any PDA (any ductal shunt detected by echocardiography); (2) hsPDA; (3) medically treated PDA; (4) surgically ligated or catheter-occluded PDA; (5) medically treated and/or surgically ligated or catheter-occluded PDA; and (6) prolonged PDA (exposure to PDA beyond 4 weeks postpartum or 36 weeks postmenstrual age). When a study reported different periods of exposure to PDA, the longest period was selected. In addition to these 6 PDA groups, PDA exposure time was collected as a continuous variable.

Methodological quality was assessed using the Newcastle-Ottawa Scale for cohort or case-control studies.^19^ Newcastle-Ottawa Scale scores of 7 or higher were considered high-quality studies (low risk of bias), and scores of 5 to 6 denoted moderate quality (moderate risk of bias).^19^

### Statistical Analysis

The effect size of dichotomous variables (PDA exposure) was expressed as log odds ratio (OR), and the effect size of continuous variables (time of exposure to PDA) was expressed using the Hedges *g* value. Values of log OR or Hedges *g* and the corresponding SEs of each individual study were calculated using Comprehensive Meta-Analysis statistical software version 4.0 (Biostat). The results were further pooled and analyzed by using bayesian model-averaged (BMA) meta-analysis.^12,13^ We performed the BMA in JASP version 0.17.3 (JASP Team 2023), which uses the metaBMA R package.^20,21^ BMA uses Bayes factors (BFs) and bayesian model averaging to evaluate the likelihood of the data under the combination of models assuming the presence vs the absence of the meta-analytic effect and heterogeneity.^12,13^ The BF_10_ is the ratio of the probability of the data under H_1_ over the probability of the data under H_0_ and was interpreted using the evidence categories suggested by Lee et al.^22^ The evidence in favor of H_1_ (BF_10_ >1) was categorized as weak or inconclusive (BF_10_ 1 to <3), moderate (BF_10_ 3 to <10), strong (BF_10_ 10 to <30), very strong (BF_10_ 30 to <100), and extreme (BF_10_ >100). The evidence in favor of H_0_ (BF_10_ <1) was categorized as weak or inconclusive (BF_10_ 1/3 to <1), moderate (BF_10_ 1/10 to <1/3), strong (BF_10_ 1/30 to <1/10), very strong (BF_10_ 1/100 to <1/30), and extreme (BF_10_ <1/100). The BF_rf_ is the ratio of the probability of the data under the random-effects model over the probability of the data under the fixed-effect model. The categories of strength of the evidence in favor of the random effects or the fixed effect were similar to those described already for BF_10_. We used the empirical prior distributions based on the Cochrane Database of Systematics Reviews transformed to log OR; log OR ~ *t* (μ = 0, σ = 0.78, ν = 5), and τ ~ inverse γ (k = 1.71, θ = 0.73).^12,13^ We used robust bayesian meta-analysis^23,24^ to assess the robustness of the results to the potential presence of publication bias.

## Results

### Description of Studies and Quality Assessment

The PRISMA flow diagram of the search process is shown in eFigure 1 in Supplement 1. Of 186 potentially relevant studies, 32 studies were included.^10,11,25,26,27,28,29,30,31,32,33,34,35,36,37,38,39,40,41,42,43,44,45,46,47,48,49,50,51,52,53,54^ These studies included 8513 infants. Characteristics of the studies are summarized in eTable 1 in Supplement 1. Risk of bias assessment according to the Newcastle-Ottawa Scale is depicted in eTable 1 in Supplement 1. All studies received a score of at least 7 points, indicating a low risk of bias. The criteria used in the various studies for the diagnosis of BPD-PH are summarized in eTable 2 in Supplement 1.

### BMA Meta-Analysis

**Figure 1.**
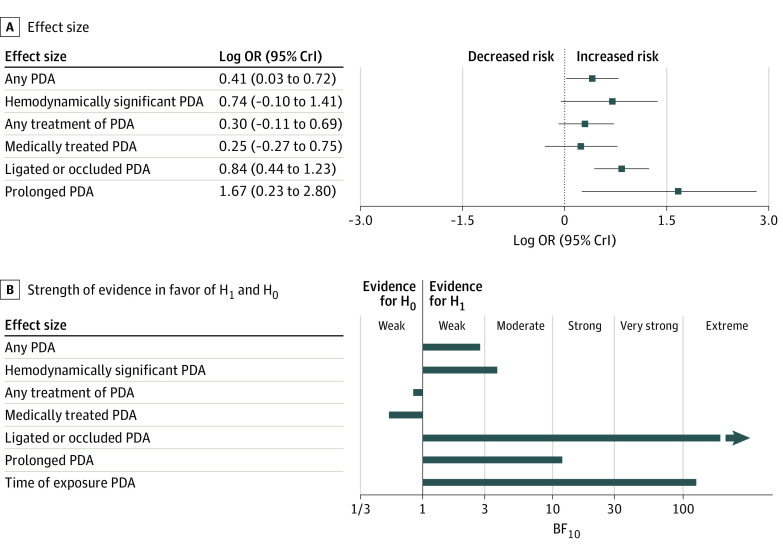
Summary of Bayesian Model-Averaged Meta-Analysis of the Association of Patent Ductus Arteriosus (PDA) With Bronchopulmonary Dysplasia–Associated Pulmonary Hypertension A, Effect sizes are shown as log odds ratios (ORs). B, Graph shows evidence in favor of the alternative hypothesis (H_1_) and the null hypothesis (H_0_) (BF_10_ denotes the ratio of the probability of the data under H_1_ over the probability of the data under H_0_). CrI indicates credible interval; OR, odds ratio.

**Figure 2.**
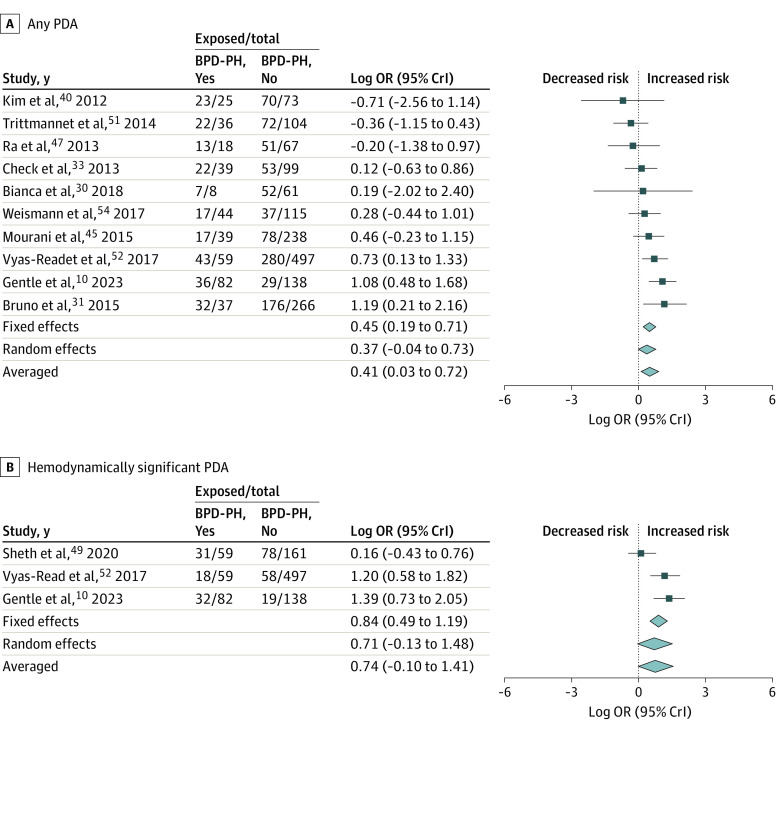
Bayesian Model Averaged Meta-Analysis of the Association of Bronchopulmonary Dysplasia-Associated Pulmonary Hypertension (BPD-PH) With Patent Ductus Arteriosus (PDA) A, Graph shows data for any PDA. B, Graph shows data for hemodynamically significant PDA. The size of the diamonds denotes the 95% credible interval (CrI). OR indicates odds ratio.

**Figure 3.**
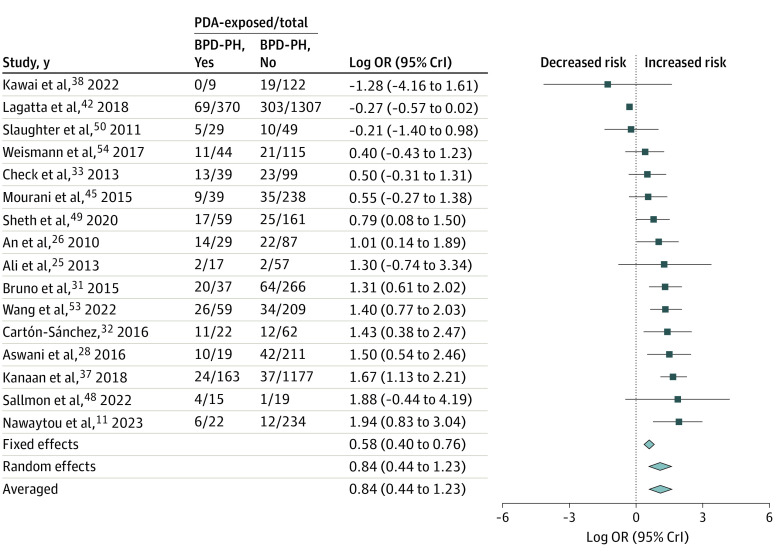
Bayesian Model-Averaged Meta-Analysis of the Association of Bronchopulmonary Dysplasia-Associated Pulmonary Hypertension (BPD-PH) With Surgically Ligated or Catheter Occluded Patent Ductus Arteriosus (PDA) The size of the diamonds denotes the 95% credible interval (CrI). OR indicates odds ratio.

**Figure 4.**
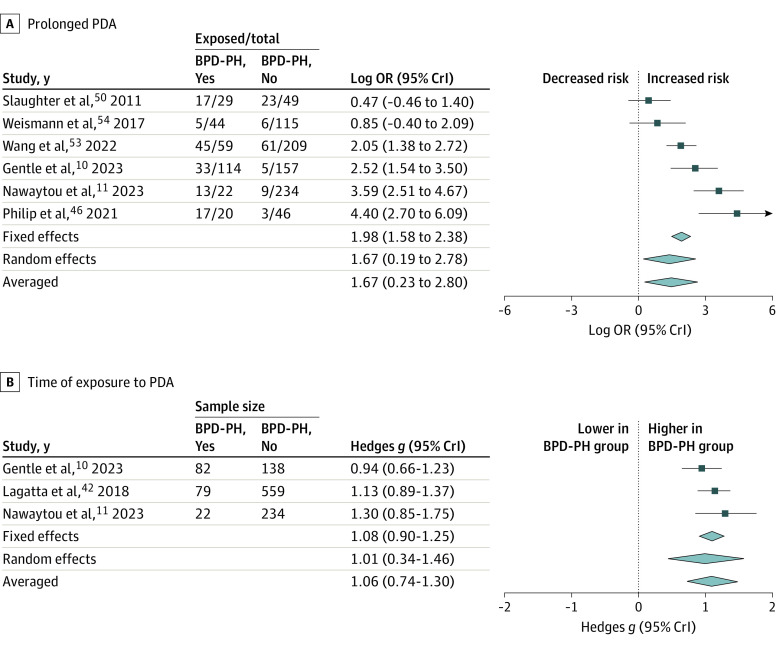
Bayesian Model-Averaged Meta-Analysis of the Association Between Bronchopulmonary Dysplasia-Associated Pulmonary Hypertension (BPD-PH) and Prolonged Exposure to Patent Ductus Arteriosus (PDA) A, Graph shows PDA as categorical variable. B, Graph shows PDA as continuous variable. The sizes of the diamonds denote the 95% credible interval (CrI). OR indicates odds ratio.

Figure 1 and the Table summarize the results of the BMA. Detailed data on heterogeneity are presented in eTable 3 in Supplement 1, and robust bayesian meta-analysis data on publication bias are shown in eTable 4 in Supplement 1. Ten studies reported on any PDA,^10,30,31,33,40,45,47,51,52,54^ and BMA showed that the evidence in favor of H_1_ (ie, the association between BPD-PH and PDA) was weak or inconclusive (BF_10_ = 2.90) (Figure 2). Three studies reported on hsPDA,^10,49,52^ and BMA showed moderate evidence in favor of H_1_ (BF_10_ = 3.77). Regarding PDA treatment, BMA showed extreme evidence (BF_10_ = 294.9; 16 studies)^11,25,26,28,31,32,33,38,42,44,45,48,49,50,53,54^ in favor of the association between BPD-PH and surgically ligated or catheter-occluded PDA (Figure 3). In contrast, BMA showed weak or inconclusive evidence in favor of H_0_ for the associations between BPD-PH and medically treated PDA (BF_10_ = 0.55; 6 studies),^10,25,32,36,38,44^ or medically treated and/or ligated or occluded PDA (BF_10_ = 0.90; 8 studies)^27,29,32,34,39,41,43,44^ (eFigure 2 in Supplement 1). Infants with BPD-PH were exposed to PDA for a pooled mean (SD) of 10.3 (2.7) days (3 studies)^10,11,42^ longer than were infants without BPD-PH. BMA showed strong evidence in favor of H_1_ when prolonged exposure to PDA was analyzed as a dichotomous variable (BF_10_ = 11.80; 6 studies)^10,11,46,50,53,54^ and extreme evidence (BF_10_ = 113.60; 3 studies)^10,11,42^ when PDA exposure time was analyzed as a continuous variable (Figure 4). Two studies^10,49^ reported effect sizes after adjustment for various covariates. The adjusted and unadjusted effect sizes are shown in eTable 5 in Supplement 1. Robust bayesian meta-analysis found no evidence for or against publication bias in any of the meta-analyses (eTable 4 in Supplement 1).

**Table.** Bayesian Model-Averaged Meta-Analysis of the Association of PDA With BPD-PH

Meta-analysis	*K* ^a^	Presence of BPD-PH, No. of infants		Log OR (SD) [95% CrI]	BF_10_^b^	Level of evidence in favor of		*P* value^c^	BF_rf_^d^	Level of evidence in favor of	
		Yes	No			H_1_	H_0_			Random effects	Fixed effects
Any PDA	10	387	1658	0.41 (0.17) [0.03 to 0.72]	2.90	Weak	NA	.02	2.20	Weak	NA
Hemodynamically meaningful PDA	3	200	796	0.74 (0.37) [−0.10 to 1.41]	3.77	Moderate	NA	.02	6.03	Moderate	NA
Medically treated PDA	6	187	1176	0.25 (0.26) [−0.27 to 0.75]	0.55	NA	Weak	.40	2.19	Weak	NA
Surgically ligated or catheter-occluded PDA	16	972	4413	0.84 (0.20) [0.44 to 1.23]	294.9	Extreme	NA	<.001	>10^6^	Extreme	NA
Medically treated and/or surgically ligated or catheter-occluded PDA	8	234	787	0.30 (0.21) [−0.11 to 0.69]	0.86	NA	Weak	.11	0.82	NA	Weak
Prolonged PDA	6	288	810	1.67 (0.65) [0.23 to 2.80]	11.80	Strong	NA	<.001	1872.5	Extreme	NA
Time of exposure to PDA	3	183	931	1.06 (0.14) [0.74 to 1.30]^e^	113.6	Extreme	NA	<.001	0.54	NA	Weak

Abbreviations: BPD-PH, bronchopulmonary dysplasia–associated pulmonary hypertension; CrI, credible interval; NA, not applicable; OR, odds ratio; PDA, patent ductus arteriosus.

^a^ *K* denotes the number of studies included in the analysis.

^b^ Bayes factor (BF)_10_ denotes the ratio of the probability of the data under the alternative hypothesis (H_1_; association of PDA with BPD-HP) over the probability of the data under the null hypothesis (H_0_).

^c^ Calculated by random effects frequentist meta-analysis.

^d^ BF_rf_ is the ratio of the probability of the data under the random-effects model over the probability of the data under the fixed-effect model.

^e^ Data are Hedges *g* values.

## Discussion

To our knowledge, this is the first bayesian meta-analysis pooling the published data on the association between PDA and BPD-PH as diagnosed by echocardiogram. Bayesian meta-analysis showed that the evidence for an association with BPD-PH was extreme for surgically treated PDA and for the continuous variable duration of PDA exposure. In addition, the evidence was moderate for hsPDA and strong for the dichotomous variable prolonged exposure to PDA. Taken together, the present data support the hypothesis that sustained patency of a hemodynamically significant ductal shunt may be a key contributor to the pathogenesis of BPD-PH.^10,11,54^

For some clinicians, PDA has acquired the status of a benign event, with treatment resulting in more complications than benefits. In the absence of a consensus on how, when, and which infants to treat for PDA, a growing number of neonatologists are adopting a less interventional approach to management of PDA.^55,56,57^ As a result, an increasing number of preterm infants are and will be potentially exposed to a prolonged ductal shunt.^58,59^ As pointed out by El-Khuffash et al,^60^ it is unlikely that a true hsPDA with chronic left-to-right shunting does not play a role in pulmonary vascular remodeling during the first few weeks of life. Accordingly, the highest evidence for an association between PDA and BPD-PH came from the group of analyses that considered the duration of ductal shunting as a continuous or categorical variable (Figure 4).

It could also be speculated that surgical ligation of PDA is a surrogate for prolonged exposure to hsPDA. This speculation is based on the fact that ligation is usually reserved for those infants in whom conservative or pharmacological treatment of PDA has failed or is contraindicated.^61^ In a recent retrospective study^62^ of a cohort of 2418 very-low-birth-weight infants who underwent invasive PDA closure, the median age at the time of the procedure was approximately 30 days. However, it cannot be overlooked that in several neonatology centers, surgical ligation of PDA has been the preferred first-line treatment for small preterm infants.^61^ Only 2 of the studies^42,49^ included in the meta-analysis provided information on the timing of invasive PDA closure. Interestingly, Lagatta et al^42^ observed that the median time at which infants underwent PDA ligation was approximately 2 weeks longer in the BPD-PH group than in the group without BPD-PH. In contrast, Sheth et al^49^ did not find any association of early (<28 days) vs late surgical closure of PDA with development of BPD-PH.

The association of PDA with BPD is complex, with a number of seemingly contradictory findings.^63^ Preclinical studies^64^ in baboons showed that exposure for 2 weeks to a moderate-to-large PDA led to a decrease in alveolar surface area and accentuated the arrest in alveolar development that characterizes BPD. However, smooth muscle abundances around terminal bronchioles and their neighboring pulmonary arteries were not affected by exposure to ductal shunt.^64^ Several recent single-center observational studies^62,64,65,66,67,68,69^ have shown that infants with small PDA shunts do not appear to be at increased risk for developing BPD. Instead, an association of PDA with BPD is apparent only when moderate-to-large shunts persist beyond 7 to 14 days.^63,65,66,67,68,69,70^ Interestingly, the duration of exposure to mechanical ventilation appears to play an important role in the interaction between PDA and BPD. Thus, Clyman et al^63,71^ showed that infants exposed to mechanical ventilation for more than 10 days developed the most severe forms of BPD, particularly when they had concurrent exposure to a moderate-to-large PDA for at least 7 to 14 days.

A reliable assessment of the impact of PDA on BPD risk requires randomized clinical trials (RCTs) in which enrollment is limited to infants with the highest risk of abnormal outcomes and in whom spontaneous ductal closure is less likely.^70^ This would ensure that ductal shunt exposure is of sufficient magnitude and duration. Conversely, the control group should achieve a high rate of ductal closure.^70^ In the last few years, several RCTs have compared early (within the first 24-72 hours after birth) pharmacologic treatment of PDA vs placebo^72,73,74,75^ or vs an expectant attitude toward PDA.^76^ However, all these RCTs focused exclusively on the incidence of BPD, without consideration of the impact of PDA on pulmonary vascular development or risk of PH. Four trials^72,73,74,75^ found no effect of early PDA treatment on the incidence of BPD, and another^76^ even reported a higher incidence of moderate-to-severe BPD in the group receiving treatment. Nevertheless, it should be noted that spontaneous ductal closure by the end of the first week occurred in as many as 20% to 50% of infants in the control group of these RCTs. In addition, ibuprofen, the drug used in most of the RCTs, closed the PDA in only 50% to 70% of the infants.^72,73,74,75,76^ Therefore, there was substantial overlap between the intervention and control groups in terms of PDA exposure.

The absence of association between a potential risk factor and BPD is not an indication that it cannot have an association with BPD-PH. The current definition of BPD, which is based on the need for supplemental oxygen and/or respiratory support at 36 weeks postmenstrual age, is an umbrella term that cannot discriminate the contribution of airway, parenchymal lung, interstitium, or pulmonary vascular disease to the respiratory difficulty.^1,3,77^ In recent years, considerable effort has been devoted to the characterization of various BPD phenotypes. Among the potential phenotypic subgroups, the so-called vascular phenotype is characterized by the associated presence of PH.^1,3,4,77^ A pathological condition such as PDA may have a specific effect on the development of the vascular phenotype of BPD without an effect on the overall incidence of BPD. An example of this was found when we recently analyzed the association between hypertensive disorders of pregnancy and BPD. Although hypertensive disorders of pregnancy were not associated with moderate-to-severe BPD, they were associated with an increased risk of developing BPD-PH.^6^

### Limitations

The main limitation of the included studies, and therefore of our meta-analysis, is the difficulty in assessing both exposure (PDA) and outcome (BPD-PH). This may account for the high heterogeneity we found in some analyses. With regard to exposure, a majority of the included studies were retrospective and did not report the time of diagnosis or treatment of PDA or the rate of response to treatment. In addition, the diagnostic criteria for hsPDA or the time limit defining prolonged exposure to PDA are the subject of ongoing and far from settled debate.^55,56,60,69,78,79,80^ It is important to note that the hemodynamic meaning of PDA is affected by factors such as the transductal diameter, the balance between pulmonary and systemic vascular resistance, and the compensatory capacity of the immature myocardium.^69^ These factors may vary over time and with clinical evolution, but this complexity is not reflected in the dichotomization of the data required by meta-analysis.

Regarding the outcome, cardiac catheterization is the criterion standard test for the diagnosis of PH, but the procedure is generally reserved for a limited group of infants because of its invasiveness.^81,82^ Echocardiography, on the other hand, has the advantage of being noninvasive and allows for serial clinical evaluation. Echocardiography screening for PH is currently recommended for infants with moderate-to-severe BPD (ie, those requiring supplemental oxygen or respiratory support at 36 weeks postmenstrual age).^83,84,85^ However, there is a lack of consensus on the echocardiography diagnostic criteria for PH in this group of infants.^85,86,87^ The echocardiography-derived measurements of PH can be classified into 3 categories: (1) indirect assessment of elevated right ventricle afterload and estimation of pulmonary hemodynamics (eg, increased tricuspid regurgitant jet velocity or flattening of the interventricular septum); (2) evaluation of measures of right and left ventricle performance; and (3) appraisal of the predominant direction of flow and degree of extrapulmonary shunts across the PDA and foramen ovale.^85,86,87^ The studies included in the present meta-analysis used different combinations of these echocardiographic measurements (see eTable 2 in Supplement 1). However, none of these individual metrics is free from criticism regarding accuracy in the diagnosis of PH and limited ability to differentiate between high flow, elevated pulmonary vascular resistance (PVR), pulmonary venous hypertension from left ventricular diastolic dysfunction, or pulmonary vein stenosis as contributors of PH.^60,85,86,87,88^

The only study included in the meta-analysis that determined the presence of PH by cardiac catheterization was Philip et al.^46^ Although that study included a very selective group of infants, its findings are very relevant to differentiate the relative role of high flow vs high PVR in BPD-PH when a ductal shunt is present. They retrospectively analyzed a series of 100 infants with GA less than 27 weeks who underwent hemodynamic evaluation before transcatheter PDA closure. In 64 infants, they observed an elevated pulmonary arterial systolic pressure (defined as pulmonary-to-systemic ratio >0.5). However, only 20 of these 64 infants had an increase in PVR. In the remaining 44 infants, the elevated pulmonary arterial systolic pressure was secondary to increased pulmonary blood flow from the PDA. Interestingly, of the 20 infants with elevated PVR, 17 had been exposed to PDA for more than 8 weeks.^46^ These data suggest that prolonged exposure to PDA often leads to pulmonary vascular disease.

## Conclusions

The findings summarized in this bayesian meta-analysis suggest that prolonged exposure to PDA may be associated with increased risk of pulmonary vascular disease in extremely preterm infants. This emphasizes the necessity of monitoring PH in high-risk preterm infants with prolonged exposure to PDA and incorporating the risk of developing PH into clinical decisions regarding definitive PDA closure by surgery or catheter occlusion. In addition, PH should be included as important outcome in clinical trials of PDA management.

## Notes

Supplement 1. **eAppendix.** Supplemental Methods **eFigure 1.** Flow Diagram of the Systematic Search **eFigure 2.** Bayesian Model Averaged Meta-Analysis on the Association Between Bronchopulmonary Dysplasia-Associated Pulmonary Hypertension and (A) Medically Treated Patent Ductus Arteriosus (PDA), and (B) Medically or Surgically Treated PDA **eTable 1.** Characteristics of the Included Studies **eTable 2.** Criteria for Echocardiographic Assessment of Pulmonary Hypertension in the Different Studies **eTable 3.** Data on Heterogeneity of the Bayesian Model-Averaged Meta-Analysis (BMA) **eTable 4.** Analysis of Publication Bias by Robust Bayesian Meta-Analysis (RoBMA) **eTable 5.** Adjusted Effect Sizes
eReferences

Supplement 2.
Data Sharing Statement

## References

[1] Thébaud B, Goss KN, Laughon M, . Bronchopulmonary dysplasia. Nat Rev Dis Primers. 2019;5(1):78. doi:10.1038/s41572-019-0127-7 31727986 PMC6986462

[2] Hansmann G, Sallmon H, Roehr CC, Kourembanas S, Austin ED, Koestenberger M; European Pediatric Pulmonary Vascular Disease Network (EPPVDN). Pulmonary hypertension in bronchopulmonary dysplasia. Pediatr Res. 2021;89(3):446-455. doi:10.1038/s41390-020-0993-4 32521539 PMC7979539

[3] Mirza H, Mandell EW, Kinsella JP, McNamara PJ, Abman SH. Pulmonary vascular phenotypes of prematurity: the path to precision medicine. J Pediatr. 2023;259:113444. doi:10.1016/j.jpeds.2023.113444 37105409 PMC10524716

[4] Durlak W, Thébaud B. The vascular phenotype of BPD: new basic science insights-new precision medicine approaches. Pediatr Res. Published online December 22, 2022. doi:10.1038/s41390-022-02428-7 36550351

[5] Nagiub M, Kanaan U, Simon D, Guglani L. Risk factors for development of pulmonary hypertension in infants with bronchopulmonary dysplasia: systematic review and meta-analysis. Paediatr Respir Rev. 2017;23:27-32. doi:10.1016/j.prrv.2016.11.003 28188008

[6] Pierro M, Villamor-Martinez E, van Westering-Kroon E, Alvarez-Fuente M, Abman SH, Villamor E. Association of the dysfunctional placentation endotype of prematurity with bronchopulmonary dysplasia: a systematic review, meta-analysis and meta-regression. Thorax. 2022;77(3):268-275. doi:10.1136/thoraxjnl-2020-216485 34301740 PMC8867288

[7] Chen Y, Zhang D, Li Y, . Risk factors and outcomes of pulmonary hypertension in infants with bronchopulmonary dysplasia: a meta-analysis. Front Pediatr. 2021;9:695610. doi:10.3389/fped.2021.695610 34249820 PMC8267150

[8] Abman SH, Shanley PF, Accurso FJ. Failure of postnatal adaptation of the pulmonary circulation after chronic intrauterine pulmonary hypertension in fetal lambs. J Clin Invest. 1989;83(6):1849-1858. doi:10.1172/JCI114091 2723062 PMC303905

[9] Reddy VM, Meyrick B, Wong J, . In utero placement of aortopulmonary shunts: a model of postnatal pulmonary hypertension with increased pulmonary blood flow in lambs. Circulation. 1995;92(3):606-613. doi:10.1161/01.CIR.92.3.606 7634475

[10] Gentle SJ, Travers CP, Clark M, Carlo WA, Ambalavanan N. Patent ductus arteriosus and development of bronchopulmonary dysplasia-associated pulmonary hypertension. Am J Respir Crit Care Med. 2023;207(7):921-928. doi:10.1164/rccm.202203-0570OC 36378949 PMC10111998

[11] Nawaytou H, Hills NK, Clyman RI. Patent ductus arteriosus and the risk of bronchopulmonary dysplasia-associated pulmonary hypertension. Pediatr Res. 2023;94(2):547-554. doi:10.1038/s41390-023-02522-4 36804505 PMC10403370

[12] Gronau QF, Heck DW, Berkhout SW, Haaf JM, Wagenmakers EJ. A primer on Bayesian model-averaged meta-analysis. Adv Methods Pract Psychol Sci. 2021;4(3):1-12. doi:10.1177/25152459211031256

[13] Bartoš F, Gronau QF, Timmers B, Otte WM, Ly A, Wagenmakers EJ. Bayesian model-averaged meta-analysis in medicine. Stat Med. 2021;40(30):6743-6761. doi:10.1002/sim.9170 34705280 PMC9298250

[14] Hoekstra R, Monden R, van Ravenzwaaij D, Wagenmakers EJ. Bayesian reanalysis of null results reported in medicine: strong yet variable evidence for the absence of treatment effects. PLoS One. 2018;13(4):e0195474. doi:10.1371/journal.pone.0195474 29694370 PMC5919013

[15] Villamor-Martinez E, Álvarez-Fuente M, Ghazi AMT, . Association of chorioamnionitis with bronchopulmonary dysplasia among preterm infants: a systematic review, meta-analysis, and metaregression. JAMA Netw Open. 2019;2(11):e1914611. doi:10.1001/jamanetworkopen.2019.14611 31693123 PMC6865274

[16] Hundscheid TM, Huizing MJ, Villamor-Martinez E, Bartoš F, Villamor E. Association of funisitis with short-term outcomes of prematurity: a frequentist and bayesian meta-analysis. Antioxidants (Basel). 2023;12(2):534. doi:10.3390/antiox12020534 36830092 PMC9951960

[17] Gonzalez-Luis GE, Borges-Lujan M, Villamor E. Association between endotypes of prematurity and pharmacological closure of patent ductus arteriosus: a systematic review and meta-analysis. Front Pediatr. 2023;11:1078506. doi:10.3389/fped.2023.1078506 36937978 PMC10020634

[18] Hundscheid TM, Villamor-Martinez E, Villamor E. Association between endotype of prematurity and mortality: a systematic review, meta-analysis, and meta-regression. Neonatology. 2023;120(4):407-416. doi:10.1159/000530127 37166331 PMC10614525

[19] Wells GA, Shea B, O’Connell D, . The Newcastle-Ottawa Scale (NOS) for assessing the quality if nonrandomized studies in meta-analyses. Accessed January 30, 2023. https://www.ohri.ca/programs/clinical_epidemiology/oxford.htm

[20] Heck D, Gronau F, Wagenmakers EJ. metaBMA: Bayesian model averaging for random and fixed effects meta-analysis, version 0.6.7. 2019. Accessed October 26, 2023. https://cran.r-project.org/web/packages/metaBMA/metaBMA.pdf

[21] van Doorn J, van den Bergh D, Böhm U, . The JASP guidelines for conducting and reporting a Bayesian analysis. Psychon Bull Rev. 2021;28(3):813-826. doi:10.3758/s13423-020-01798-5 33037582 PMC8219590

[22] Lee M, Wagenmakers EJ. Bayesian Data Analysis for Cognitive Science: A Practical Course. Cambridge University Press; 2013.

[23] Maier M, Bartoš F, Wagenmakers EJ. Robust Bayesian meta-analysis: addressing publication bias with model-averaging. Psychol Methods. 2023;28(1):107-122. doi:10.1037/met0000405 35588075

[24] Bartoš F, Maier M, Wagenmakers EJ, Doucouliagos H, Stanley TD. Robust Bayesian meta-analysis: model-averaging across complementary publication bias adjustment methods. Res Synth Methods. 2023;14(1):99-116. doi:10.1002/jrsm.1594 35869696 PMC10087723

[25] Ali Z, Schmidt P, Dodd J, Jeppesen DL. Predictors of bronchopulmonary dysplasia and pulmonary hypertension in newborn children. Dan Med J. 2013;60(8):A4688. 23905570

[26] An HS, Bae EJ, Kim GB, . Pulmonary hypertension in preterm infants with bronchopulmonary dysplasia. Korean Circ J. 2010;40(3):131-136. doi:10.4070/kcj.2010.40.3.131 20339498 PMC2844979

[27] Arattu Thodika FMS, Nanjundappa M, Dassios T, Bell A, Greenough A. Pulmonary hypertension in infants with bronchopulmonary dysplasia: risk factors, mortality and duration of hospitalisation. J Perinat Med. 2021;50(3):327-333. doi:10.1515/jpm-2021-0366 34847313

[28] Aswani R, Hayman L, Nichols G, . Oxygen requirement as a screening tool for the detection of late pulmonary hypertension in extremely low birth weight infants. Cardiol Young. 2016;26(3):521-527. doi:10.1017/S1047951115000608 26119883

[29] Bhat R, Salas AA, Foster C, Carlo WA, Ambalavanan N. Prospective analysis of pulmonary hypertension in extremely low birth weight infants. Pediatrics. 2012;129(3):e682-e689. doi:10.1542/peds.2011-1827 22311993 PMC3289526

[30] Blanca AJ, Duijts L, van Mastrigt E, . Right ventricular function in infants with bronchopulmonary dysplasia and pulmonary hypertension: a pilot study. Pulm Circ. Published online December 1, 2018. doi:10.1177/2045894018816063 30419798 PMC6295707

[31] Bruno CJ, Meerkov M, Capone C, . CRIB scores as a tool for assessing risk for the development of pulmonary hypertension in extremely preterm infants with bronchopulmonary dysplasia. Am J Perinatol. 2015;32(11):1031-1037. doi:10.1055/s-0035-1547324 26368789

[32] Cartón Sánchez AJ. Hipertensión pulmonar estimada por ecocardiografía en prematuros con displasia broncopulmonar: frecuencia, evolución y factores de riesgo. 2016. Accessed January 30, 2023. https://repositorio.uam.es/handle/10486/674863

[33] Check J, Gotteiner N, Liu X, . Fetal growth restriction and pulmonary hypertension in premature infants with bronchopulmonary dysplasia. J Perinatol. 2013;33(7):553-557. doi:10.1038/jp.2012.164 23328924 PMC3633609

[34] Choi EK, Jung YH, Kim HS, . The impact of atrial left-to-right shunt on pulmonary hypertension in preterm infants with moderate or severe bronchopulmonary dysplasia. Pediatr Neonatol. 2015;56(5):317-323. doi:10.1016/j.pedneo.2014.12.006 26328892

[35] Dasgupta S, Aly AM, Malloy MH, Okorodudu AO, Jain SK. NTproBNP as a surrogate biomarker for early screening of pulmonary hypertension in preterm infants with bronchopulmonary dysplasia. J Perinatol. 2018;38(9):1252-1257. doi:10.1038/s41372-018-0164-1 29977013

[36] DeVries LB, Heyne RJ, Ramaciotti C, . Mortality among infants with evolving bronchopulmonary dysplasia increases with major surgery and with pulmonary hypertension. J Perinatol. 2017;37(9):1043-1046. doi:10.1038/jp.2017.89 28617427

[37] Kanaan U, Srivatsa B, Huckaby J, Kelleman M. Association of unit-wide oxygen saturation target on incidence of pulmonary hypertension in very low birthweight premature infants. J Perinatol. 2018;38(2):148-153. doi:10.1038/jp.2017.166 29048404

[38] Kawai Y, Hayakawa M, Tanaka T, . Pulmonary hypertension with bronchopulmonary dysplasia: Aichi cohort study. Pediatr Int. 2022;64(1):e15271. doi:10.1111/ped.15271 35972055

[39] Khemani E, McElhinney DB, Rhein L, . Pulmonary artery hypertension in formerly premature infants with bronchopulmonary dysplasia: clinical features and outcomes in the surfactant era. Pediatrics. 2007;120(6):1260-1269. doi:10.1542/peds.2007-0971 18055675

[40] Kim DH, Kim HS, Choi CW, Kim EK, Kim BI, Choi JH. Risk factors for pulmonary artery hypertension in preterm infants with moderate or severe bronchopulmonary dysplasia. Neonatology. 2012;101(1):40-46. doi:10.1159/000327891 21791938

[41] Kunjunju AM, Gopagondanahalli KR, Chan Y, Sehgal A. Bronchopulmonary dysplasia-associated pulmonary hypertension: clues from placental pathology. J Perinatol. 2017;37(12):1310-1314. doi:10.1038/jp.2017.130 28880261

[42] Lagatta JM, Hysinger EB, Zaniletti I, . The impact of pulmonary hypertension in preterm infants with severe bronchopulmonary dysplasia through 1 year. J Pediatr. 2018;203:218-224.e3. doi:10.1016/j.jpeds.2018.07.035 30172426 PMC6460906

[43] Lodha A, Thomas S, Jain S, . Neurodevelopmental outcomes of preterm infants born <29 weeks with bronchopulmonary dysplasia associated pulmonary hypertension: a multicenter study. *Res Sq*. Preprint posted online August 22, 2022. doi:10.21203/rs.3.rs-1956482/v1 37399847

[44] Madden BA, Conaway MR, Zanelli SA, McCulloch MA. Screening echocardiography identifies risk factors for pulmonary hypertension at discharge in premature infants with bronchopulmonary dysplasia. Pediatr Cardiol. 2022;43(8):1743-1751. doi:10.1007/s00246-022-02911-2 35488130

[45] Mourani PM, Sontag MK, Younoszai A, . Early pulmonary vascular disease in preterm infants at risk for bronchopulmonary dysplasia. Am J Respir Crit Care Med. 2015;191(1):87-95. doi:10.1164/rccm.201409-1594OC 25389562 PMC4299632

[46] Philip R, Waller BR, Chilakala S, . Hemodynamic and clinical consequences of early versus delayed closure of patent ductus arteriosus in extremely low birth weight infants. J Perinatol. 2021;41(1):100-108. doi:10.1038/s41372-020-00772-2 32792636

[47] Ra JJ, Lee SM, Eun HS, . Risk factors of pulmonary hypertension in preterm infants with chronic lung disease. Neonatal Med. 2013;20(1):75-80. doi:10.5385/nm.2013.20.1.75

[48] Sallmon H, Koestenberger M, Avian A, . Extremely premature infants born at 23-25 weeks gestation are at substantial risk for pulmonary hypertension. J Perinatol. 2022;42(6):781-787. doi:10.1038/s41372-022-01374-w 35365772 PMC9184271

[49] Sheth S, Goto L, Bhandari V, Abraham B, Mowes A. Factors associated with development of early and late pulmonary hypertension in preterm infants with bronchopulmonary dysplasia. J Perinatol. 2020;40(1):138-148. doi:10.1038/s41372-019-0549-9 31723236 PMC7223406

[50] Slaughter JL, Pakrashi T, Jones DE, South AP, Shah TA. Echocardiographic detection of pulmonary hypertension in extremely low birth weight infants with bronchopulmonary dysplasia requiring prolonged positive pressure ventilation. J Perinatol. 2011;31(10):635-640. doi:10.1038/jp.2010.213 21311503

[51] Trittmann JK, Nelin LD, Zmuda EJ, . Arginase I gene single-nucleotide polymorphism is associated with decreased risk of pulmonary hypertension in bronchopulmonary dysplasia. Acta Paediatr. 2014;103(10):e439-e443. doi:10.1111/apa.12717 24919409 PMC4180790

[52] Vyas-Read S, Kanaan U, Shankar P, . Early characteristics of infants with pulmonary hypertension in a referral neonatal intensive care unit. BMC Pediatr. 2017;17(1):163. doi:10.1186/s12887-017-0910-0 28697724 PMC5506674

[53] Wang C, Ma X, Xu Y, Chen Z, Shi L, Du L. A prediction model of pulmonary hypertension in preterm infants with bronchopulmonary dysplasia. Front Pediatr. 2022;10:925312. doi:10.3389/fped.2022.925312 35935371 PMC9354604

[54] Weismann CG, Asnes JD, Bazzy-Asaad A, Tolomeo C, Ehrenkranz RA, Bizzarro MJ. Pulmonary hypertension in preterm infants: results of a prospective screening program. J Perinatol. 2017;37(5):572-577. doi:10.1038/jp.2016.255 28206997

[55] Hamrick SEG, Sallmon H, Rose AT, . Patent ductus arteriosus of the preterm infant. Pediatrics. 2020;146(5):e20201209. doi:10.1542/peds.2020-1209 33093140 PMC7605084

[56] Hundscheid T, El-Khuffash A, McNamara PJ, de Boode WP. Survey highlighting the lack of consensus on diagnosis and treatment of patent ductus arteriosus in prematurity. Eur J Pediatr. 2022;181(6):2459-2468. doi:10.1007/s00431-022-04441-8 35305143 PMC9110525

[57] Hagadorn JI, Brownell EA, Trzaski JM, . Trends and variation in management and outcomes of very low-birth-weight infants with patent ductus arteriosus. Pediatr Res. 2016;80(6):785-792. doi:10.1038/pr.2016.166 27509008

[58] Tolia VN, Powers GC, Kelleher AS, . Low rate of spontaneous closure in premature infants discharged with a patent ductus arteriosus: a multicenter prospective study. J Pediatr. 2022;240:31-36.e2. doi:10.1016/j.jpeds.2021.07.035 34293369

[59] Bischoff AR, Cavallaro Moronta S, McNamara PJ. Going home with a patent ductus arteriosus: is it benign? J Pediatr. 2022;240:10-13. doi:10.1016/j.jpeds.2021.09.009 34530023

[60] El-Khuffash A, Mullaly R, McNamara PJ. Patent ductus arteriosus, bronchopulmonary dysplasia and pulmonary hypertension—a complex conundrum with many phenotypes? Pediatr Res. 2023;94(2):416-417. doi:10.1038/s41390-023-02578-2 36934215 PMC10382312

[61] Reese J, Scott TA, Patrick SW. Changing patterns of patent ductus arteriosus surgical ligation in the United States. Semin Perinatol. 2018;42(4):253-261. doi:10.1053/j.semperi.2018.05.008 29954594 PMC6512985

[62] Lai KC, Richardson T, Berman D, . Current trends in invasive closure of patent ductus arteriosus in very low birth weight infants in United States children’s hospitals, 2016-2021. J Pediatr. 2023;263:113712. doi:10.1016/j.jpeds.2023.113712 37659587

[63] Clyman RI, Hills NK. Patent ductus arteriosus (PDA) and pulmonary morbidity: can early targeted pharmacologic PDA treatment decrease the risk of bronchopulmonary dysplasia? Semin Perinatol. 2023;47(2):151718. doi:10.1016/j.semperi.2023.151718 36882361

[64] McCurnin D, Seidner S, Chang LY, . Ibuprofen-induced patent ductus arteriosus closure: physiologic, histologic, and biochemical effects on the premature lung. Pediatrics. 2008;121(5):945-956. doi:10.1542/peds.2007-2051 18450898 PMC11790498

[65] Schena F, Francescato G, Cappelleri A, . Association between hemodynamically significant patent ductus arteriosus and bronchopulmonary dysplasia. J Pediatr. 2015;166(6):1488-1492. doi:10.1016/j.jpeds.2015.03.012 25882876

[66] Sellmer A, Bjerre JV, Schmidt MR, . Morbidity and mortality in preterm neonates with patent ductus arteriosus on day 3. Arch Dis Child Fetal Neonatal Ed. 2013;98(6):F505-F510. doi:10.1136/archdischild-2013-303816 23893268

[67] Clyman RI, Hills NK, Liebowitz M, Johng S. Relationship between duration of infant exposure to a moderate-to-large patent ductus arteriosus shunt and the risk of developing bronchopulmonary dysplasia or death before 36 weeks. Am J Perinatol. 2020;37(2):216-223. doi:10.1055/s-0039-1697672 31600791 PMC9940607

[68] Mirza H, Garcia J, McKinley G, . Duration of significant patent ductus arteriosus and bronchopulmonary dysplasia in extremely preterm infants. J Perinatol. 2019;39(12):1648-1655. doi:10.1038/s41372-019-0496-5 31554913

[69] Clyman RI, Hills NK. The effect of prolonged tracheal intubation on the association between patent ductus arteriosus and bronchopulmonary dysplasia (grades 2 and 3). J Perinatol. 2020;40(9):1358-1365. doi:10.1038/s41372-020-0718-x 32669644 PMC7442702

[70] Deng Y, Zhang H, Zhao Z, Du J, Bai R, McNamara PJ. Impact of patent ductus arteriosus shunt size and duration on risk of death or severe respiratory morbidity in preterm infants born in China. Eur J Pediatr. 2022;181(8):3131-3140. doi:10.1007/s00431-022-04549-x 35838780 PMC9352633

[71] Clyman RI, Kaempf J, Liebowitz M, . Prolonged tracheal intubation and the association between patent ductus arteriosus and bronchopulmonary dysplasia: a secondary analysis of the PDA-TOLERATE trial. J Pediatr. 2021;229:283-288.e2. doi:10.1016/j.jpeds.2020.09.047 32979387 PMC7855529

[72] Gupta S, Juszczak E, Subhedar N, . Does selective early treatment of patent ductus arteriosus (PDA) with ibuprofen reduce death or bronchopulmonary dysplasia (BPD) at 36 weeks in extreme preterm babies? a randomised controlled trial (Baby-OSCAR Trial). Arch Dis Child. 2022;107(suppl 2):545. doi:10.1136/archdischild-2022-rcpch.241

[73] Rozé J-C, Cambonie G, Le Thuaut A, . Effect of early targeted treatment of ductus arteriosus with ibuprofen on survival without cerebral palsy at 2 years in infants with extreme prematurity: a randomized clinical trial. J Pediatr. 2021;233:33-42.e2. doi:10.1016/j.jpeds.2020.12.008 33307111

[74] El-Khuffash A, Bussmann N, Breatnach CR, . A pilot randomized controlled trial of early targeted patent ductus arteriosus treatment using a risk based severity score (the PDA RCT). J Pediatr. 2021;229:127-133. doi:10.1016/j.jpeds.2020.10.024 33069668

[75] de Waal K, Phad N, Stubbs M, Chen Y, Kluckow M. A randomized placebo-controlled pilot trial of early targeted nonsteroidal anti-inflammatory drugs in preterm infants with a patent ductus arteriosus. J Pediatr. 2021;228:82-86.e2. doi:10.1016/j.jpeds.2020.08.062 32858033

[76] Hundscheid T, Onland W, Kooi EM, . Expectant management or early ibuprofen for patent ductus arteriosus. N Engl J Med. 2023;388(11):980-990. doi:10.1056/NEJMoa2207418 36477458

[77] Pierro M, Van Mechelen K, van Westering-Kroon E, Villamor-Martínez E, Villamor E. Endotypes of prematurity and phenotypes of bronchopulmonary dysplasia: toward personalized neonatology. J Pers Med. 2022;12(5):687. doi:10.3390/jpm12050687 35629108 PMC9143617

[78] Philip R, Lamba V, Talati A, Sathanandam S. Pulmonary hypertension with prolonged patency of the ductus arteriosus in preterm infants. Children (Basel). 2020;7(9):139. doi:10.3390/children7090139 32947808 PMC7552711

[79] Haregu F, McCulloch M, Vergales B, Garrod A, Conaway M, Hainstock M. Transcatheter occlusion of left-to-right shunts in premature infants with bronchopulmonary dysplasia. Neonatology. 2023;120(1):57-62. doi:10.1159/000527267 36516787

[80] Sehgal A, McNamara PJ. Does echocardiography facilitate determination of hemodynamic significance attributable to the ductus arteriosus? Eur J Pediatr. 2009;168(8):907-914. doi:10.1007/s00431-009-0983-3 19387684

[81] Dasgupta S, Richardson JC, Aly AM, Jain SK. Role of functional echocardiographic parameters in the diagnosis of bronchopulmonary dysplasia-associated pulmonary hypertension. J Perinatol. 2022;42(1):19-30. doi:10.1038/s41372-021-01009-6 33686118 PMC7938691

[82] Frank BS, Schäfer M, Grenolds A, Ivy DD, Abman SH, Darst JR. Acute vasoreactivity testing during cardiac catheterization of neonates with bronchopulmonary dysplasia-associated pulmonary hypertension. J Pediatr. 2019;208:127-133. doi:10.1016/j.jpeds.2018.12.004 30871795

[83] Abman SH, Hansmann G, Archer SL, ; American Heart Association Council on Cardiopulmonary, Critical Care, Perioperative and Resuscitation; Council on Clinical Cardiology; Council on Cardiovascular Disease in the Young; Council on Cardiovascular Radiology and Intervention; Council on Cardiovascular Surgery and Anesthesia; and the American Thoracic Society. Pediatric pulmonary hypertension: guidelines from the American heart association and American thoracic Society. Circulation. 2015;132(21):2037-2099. doi:10.1161/CIR.0000000000000329 26534956

[84] Levin JC, Annesi CA, Williams DN, . Discharge practices for infants with bronchopulmonary dysplasia: a survey of national experts. J Pediatr. 2023;253:72-78.e3. doi:10.1016/j.jpeds.2022.09.018 36126730 PMC10423686

[85] Krishnan U, Feinstein JA, Adatia I, . Evaluation and management of pulmonary hypertension in children with bronchopulmonary dysplasia. J Pediatr. 2017;188:24-34.e1. doi:10.1016/j.jpeds.2017.05.029 28645441

[86] Nagiub M, Lee S, Guglani L. Echocardiographic assessment of pulmonary hypertension in infants with bronchopulmonary dysplasia: systematic review of literature and a proposed algorithm for assessment. Echocardiography. 2015;32(5):819-833. doi:10.1111/echo.12738 25231322

[87] Levy PT, Levin J, Leeman KT, Mullen MP, Hansmann G, Kourembanas S. Diagnosis and management of pulmonary hypertension in infants with bronchopulmonary dysplasia. Semin Fetal Neonatal Med. 2022;27(4):101351. doi:10.1016/j.siny.2022.101351 35641413

[88] McNamara PJ, Lakshminrusimha S. Maldevelopment of the immature pulmonary vasculature and prolonged patency of the ductus arteriosus: association or cause? Am J Respir Crit Care Med. 2023;207(7):814-816. doi:10.1164/rccm.202211-2146ED 36470238 PMC10111972

