# Supplementary material for: Patent Ductus Arteriosus and Bronchopulmonary Dysplasia–Associated Pulmonary Hypertension: A Bayesian Meta-Analysis
Source: JAMA Netw Open. 2023 Nov 28;6(11):e2345299. doi: 10.1001/jamanetworkopen.2023.45299 (PMC10685885; doi:10.1001/jamanetworkopen.2023.45299)
Supplement: Supplement 1. — eAppendix. Supplemental Methods eFigure 1. Flow Diagram of the Systematic Search eFigure 2. Bayesian Model Averaged Meta-Analysis on the Association Between Bronchopulmonary Dysplasia-Associated Pulmonary Hypertension and (A) Medically Treated Patent Ductus Arteriosus (PDA), and (B) Medically or Surgically Treated PDA eTable 1. Characteristics of the Included Studies eTable 2. Criteria for Echocardiographic Assessment of Pulmonary Hypertension in the Different Studies eTable 3. Data on Heterogeneity of the Bayesian Model-Averaged Meta-Analysis (BMA) eTable 4. Analysis of Publication Bias by Robust Bayesian Meta-Analysis (RoBMA) eTable 5. Adjusted Effect Sizes eReferences [file jamanetwopen-e2345299-s001.pdf]

Supplemental Online Content

Villamor E, van Westering-Kroon E, Gonzalez-Luis GE, Bartoš F, Abman SH, Huizing MJ. Patent ductus arteriosus and bronchopulmonary dysplasia–associated pulmonary hypertension: a bayesian meta-analysis. *JAMA Netw Open*. 2023;6(11):e2345299. doi:10.1001/jamanetworkopen.2023.45299

eAppendix 1. Supplemental Methods

eAppendix 2. Supplemental Results

eFigure 1. Flow Diagram of the Systematic Search

eFigure 2. Bayesian Model Averaged Meta-Analysis on the Association Between Bronchopulmonary Dysplasia-Associated Pulmonary Hypertension and (A) Medically Treated Patent Ductus Arteriosus (PDA), and (B) Medically or Surgically Treated PDA

eTable 1. Characteristics of the Included Studies

eTable 2. Criteria for Echocardiographic Assessment of Pulmonary Hypertension in the Different Studies

eTable 3. Data on Heterogeneity of the Bayesian Model-Averaged Meta-Analysis (BMA)

eTable 4. Analysis of Publication Bias by Robust Bayesian Meta-Analysis (RoBMA)

eTable 5. Adjusted Effect Sizes

eReferences

This supplemental material has been provided by the authors to give readers additional information about their work.

eAppendix 1. Supplemental Methods

1.1. Search strategy

Pubmed

(pulmonary hypertension [MESH] OR pulmonary hypertension [tiab])<br>AND<br>(bronchopulmonary dysplasia [MESH] OR bronchopulmonary dysplasia [tiab] OR BPD [tiab] OR chronic lung disease [tiab] OR CLD [tiab] OR ductus arteriosus [MESH] OR ductus arteriosus [tiab] OR PDA [tiab])<br>AND<br>(preterm infant [tiab] OR Premature Infant [tiab] OR Premature Infants [tiab] OR preterm infants [tiab] OR neonatal prematurity [tiab] OR very low birth weight infant [tiab] OR Very-Low-Birth-Weight Infant [tiab] OR Very-Low-Birth-Weight Infants [tiab] OR very low birth weight infants [tiab] OR Extremely Low Birth Weight Infant [tiab] OR Extremely Low Birth Weight Infants [tiab] OR preterm infant [MESH] OR Premature Infant [MESH] OR Premature Infants [MESH] OR preterm infants [MESH] OR neonatal prematurity [MESH] OR very low birth weight infant [MESH] OR Very-Low-Birth-Weight Infant [MESH] OR Very-Low-Birth-Weight Infants [MESH] OR very low birth weight infants [MESH] OR Extremely Low Birth Weight Infant [MESH] OR Extremely Low Birth Weight Infants)

EMBASE

('chronic lung disease'/exp OR 'chronic lung disease') AND (‘pulmonary hypertension’/exp OR ‘pulmonary hypertension’) AND ('ductus arteriosus'/exp OR 'ductus arteriosus') AND (premature infant or Neonatal Prematurity or Infants, Premature or Prematurity or Neonatal or Preterm Infants)

Web of Science

((bronchopulmonary dysplasia OR BPD OR chronic lung disease) AND (“pulmonary hypertension”) AND ("ductus arteriosus" OR PDA))

No language limits were set. Narrative reviews, systematic reviews, case reports, letters, editorials, and commentaries were excluded, but read to identify potential additional studies. Additional strategies to identify studies included manual review of reference lists from key articles that fulfilled our eligibility criteria, use of “related articles” feature in PubMed, and use of the “cited by” tool in Web of Science and Google scholar. Two reviewers independently screened the results of the searches, and included studies according to the inclusion criteria using EndNote (RRID:SCR_014001), using the methodology described by Bramer et al.[1]

1.2 Robust Bayesian meta-analysis (RoBMA)

We used RoBMA to assess the robustness of the results to the potential presence of publication bias.[2,3] RoBMA extends the Bayesian model-averaged meta-analysis by the two major publication bias adjustment techniques: selection models (adjusting for the publication bias operating on p-values)[4] and precision-effect test and precision-effect estimate with standard errors (PET-PEESE, adjusting for the relationship between effect sizes and standard errors).[5] The resulting RoBMA ensemble contains 36 models composed of the following assumptions about the presence vs. absence of the effect (2) x presence vs. absence of between-study heterogeneity (2) x presence vs. absence of publication bias adjustment models (6 selection models, PET, PEESE, and no bias). We used RoBMA with the same prior distributions for the effect and heterogeneity as in BMA and the default prior distributions for the publication bias adjustment part.

© 2023 Villamor E et al. *JAMA Network Open.*

eAppendix 2. Supplemental Results

2.1. Supplementary Figures

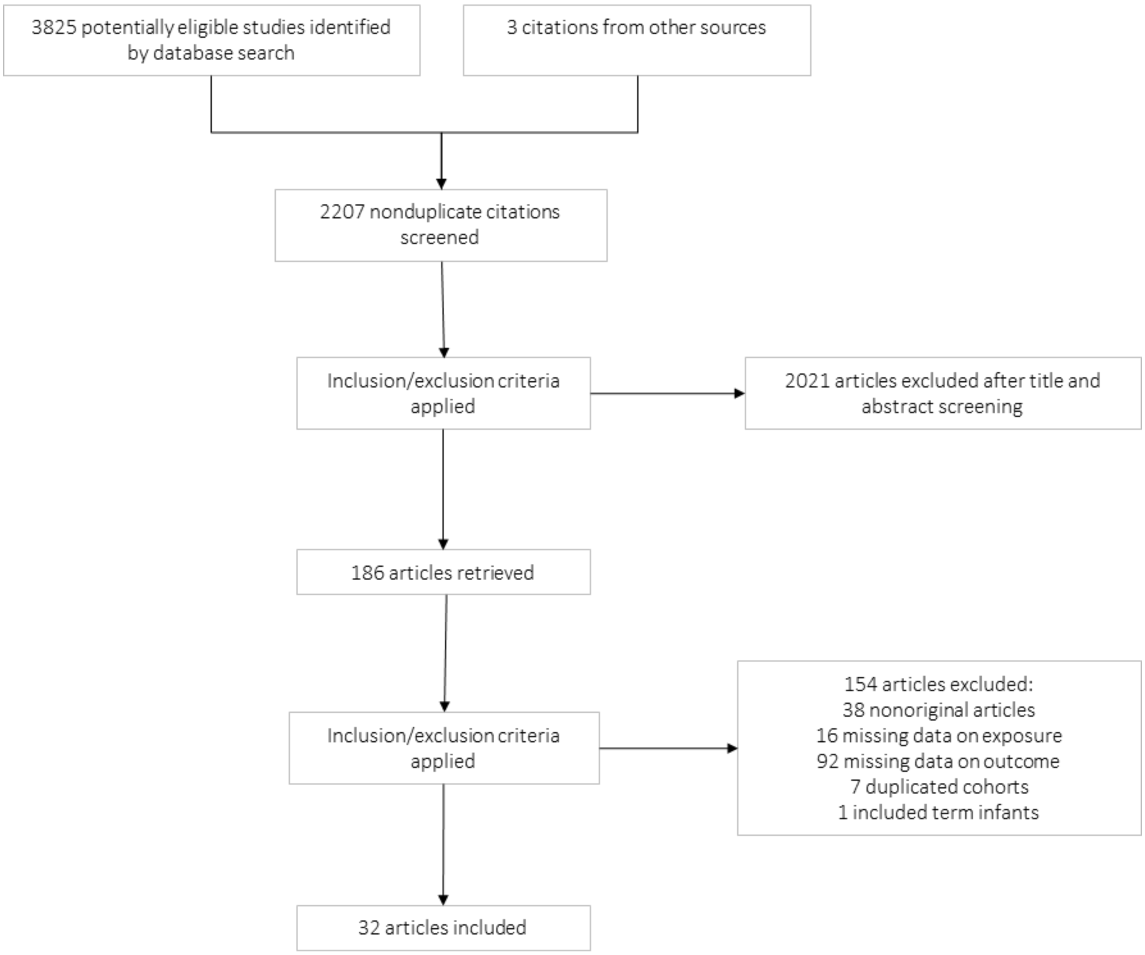

eFigure 1. Flow Diagram of the Systematic Search

© 2023 Villamor E et al. *JAMA Network Open.*

A

Medically treated PDA (k=6)

Study name	Exposed/ Total BPD-PH Yes	Exposed/ Total BPD-PH No	Statistics for each study Log odds ratio	Lower limit	Upper limit
DeVries et al,[17] 2017	5 / 18	284 / 559	-0.99	-2.03	0.06
Kawai et al,[20] 2022	4 / 9	63 / 122	-0.29	-1.65	1.07
Mourani et al,[27] 2015	17 / 39	101 / 238	0.05	-0.64	0.73
Gentle et al,[18] 2023	20 / 82	19 / 138	0.70	0.00	1.40
Ali et al,[6] 2013	9 / 17	20 / 57	0.73	-0.36	1.83
Cartón-Sánchez,[13] 2016	18 / 22	33 / 62	1.37	0.18	2.57
**Fixed effects**			**0.27**	**-0.09**	**0.62**
**Random effects**			**0.23**	**-0.33**	**0.79**
**Averaged**			**0.25**	**-0.27**	**0.75**

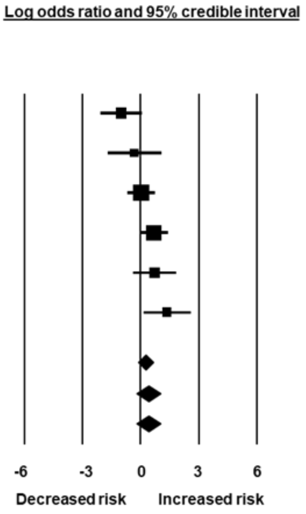

B

Any treatment of PDA (k=8)

Study name	Exposed/ Total BPD-PH Yes	Exposed/ Total BPD-PH No	Statistics for each study Log odds ratio	Lower limit	Upper limit
Khemani et al,[21] 2007	10 / 18	16 / 24	-0.47	-1.73	0.79
Kunjunju et al,[23] 2017	10 / 22	18 / 34	-0.30	-1.38	0.78
Bhat et al,[10] 2012	3 / 26	17 / 119	-0.25	-1.55	1.06
Madden et al,[26] 2022	7 / 22	15 / 42	-0.17	-1.27	0.92
Lodha et al,[25] 2022	37 / 52	120 / 202	0.52	-0.14	1.18
Arattu et al,[8] 2021	12 / 22	61 / 160	0.67	-0.23	1.56
Choi et al,[15] 2015	48 / 50	129 / 144	1.03	-0.49	2.54
Cartón-Sánchez,[13] 2016	18 / 22	33 / 62	1.37	0.18	2.57
**Fixed effects**			**0.31**	**-0.03**	**0.66**
**Random effects**			**0.28**	**-0.20**	**0.73**
**Averaged**			**0.30**	**-0.11**	**0.69**

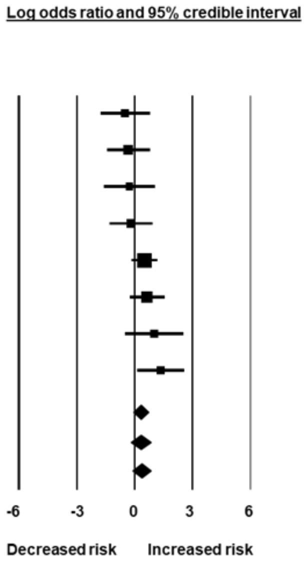

eFigure 2. Bayesian Model Averaged Meta-Analysis on the Association Between Bronchopulmonary Dysplasia-Associated Pulmonary Hypertension and (A) Medically Treated Patent Ductus Arteriosus (PDA), and (B) Medically or Surgically Treated PDA

© 2023 Villamor E et al. *JAMA Network Open.*

2.2. Supplementary Tables

eTable 1. Characteristics of the Included Studies

First author, year	Country	Prospective?	Total infants	Centers	GA of cohort (weeks)	PDA group	NOS Selection	NOS Comparability	NOS Outcome/Exp.	NOS Total
Ali, 2013[6]	Denmark	No	392	1	26.7	MedPDA SurgPDA	4	2	3	9
An, 2010 [7]	Korea	No	116	1	26.3	SurgPDA	4	1	3	8
Arattu, 2021[8]	UK	No	182	1	25.9	TreatPDA	4	1	3	8
Aswani, 2016 [9]	USA	No	230	1	25.9	SurgPDA	4	1	3	8
Bhat, 2012 [10]	USA	Yes	145	1	26	TreatPDA	4	2	3	9
Blanca, 2018 [11]	NL	Yes	69	1	25.6	Any PDA	3	1	3	7
Bruno, 2015 [12]	USA	No	303	1	26.6	Any PDA SurgPDA	4	2	3	9
Cartón, 2016 [13]	Spain	Yes	84	1	27.0	MedPDA SurgPDA TreatPDA	4	2	3	9
Check, 2013 [14]	USA	No	138	1	26.1	Any PDA SurgPDA	3	1	3	7
Choi, 2015 [15]	Korea	No	194	1	26.5	TreatPDA	4	1	3	8
Dasgupta, 2018 [16]	USA	Yes	36	1	26.6	HsPDA MedPDA	4	2	3	9
DeVries, 2017 [17]	USA	No	577	1	26.6	MedPDA	4	2	2	8
Gentle, 2023 [18]	USA	Yes	220	1	25.7	Any PDA HsPDA	4	2	3	9

© 2023 Villamor E et al. *JAMA Network Open.*

First author, year	Country	Prospective?	Total infants	Centers	GA of cohort (weeks)	PDA group	NOS Selection	NOS Comparability	NOS Outcome/Exp.	NOS Total
						MedPDA ProlPDA TimePDA				
Kanaan, 2018 [19]	USA	No	1340	1	27.8	SurgPDA	4	2	3	9
Kawai, 2022 [20]	Japan	No	131	1	26.0	MedPDA SurgPDA	4	2	3	9
Khemani, 2007 [21]	USA	No	42	3	26.0	TreatPDA	3	1	3	7
Kim, 2012 [22]	Korea	No	98	1	26.8	Any PDA	4	1	3	8
Kunjunju, 2017 [23]	Australia	No	56	1	26.0	TreatPDA	3	1	3	7
Lagatta, 2018 [24]	USA	No	1677	23	25.0	SurgPDA TimePDA	3	2	3	8
Lodha, 2022 [25]	USA	No	254	1	25.8	TreatPDA	3	2	3	8
Madden 2022 [26]	USA	No	64	1	26.2	TreatPDA	4	1	3	8
Mourani, 2015 [27]	USA	Yes	274	2	27.0	Any PDA MedPDA SurgPDA	4	1	3	8
Nawaytou, 2022[28]	USA	Yes	256	1	26.2	SurgPDA ProlPDA TimePDA	4	1	3	8
Philip 2021[29]	USA	No	100	1	24	ProlPDA	3	1	3	7
Ra, 2013 [30]	Korea	No	85	1	28.0	Any PDA	3	1	3	7

© 2023 Villamor E et al. *JAMA Network Open.*

First author, year	Country	Prospective?	Total infants	Centers	GA of cohort (weeks)	PDA group	NOS Selection	NOS Comparability	NOS Outcome/Exp.	NOS Total
Sallmon, 2022[31]	Germany	Yes	34	1	24.7	SurgPDA	4	2	3	9
Sheth, 2020 [32]	USA	No	220	1	25.9	HsPDA<br>SurgPDA	4	2	3	9
Slaughter, 2011[33]	USA	No	78	3	25	SurgPDA<br>ProlPDA	4	2	2	8
Trittmann, 2014[34]	USA	Yes	140	1	28	Any PDA	4	2	3	9
Vyas-Read, 2017[35]	USA	No	556	2	26.1	Any PDA<br>HsPDA	4	2	3	9
Wang, 2022 [36]	China	no	268	1	28.2	SurgPDA<br>ProlPDA	4	1	3	8
Weismann, 2017 [37]	USA	Yes	159	1	25.6	AnyPDA<br>SurgPDA<br>ProlPDA	4	2	3	9

AnyPDA: any ductal shunt detected by echocardiography; HsPDA: hemodynamically significant PDA; MedPDA: medically treated PDA; SurgPDA: surgically-ligated or catheter-occludded PDA; TreatPDA: medically treated and/or surgically ligated/catheter occluded PDA; ProlPDA: exposure to PDA beyond 4 weeks postpartum or 36 weeks postmenstrual age. TimePDA: time of exposure to PDA.

© 2023 Villamor E et al. *JAMA Network Open.*

eTable 2. Criteria for Echocardiographic Assessment of Pulmonary Hypertension in the Different Studies

Study	Age at echocardiography	Criteria for echocardiographic assessment of pulmonay hypertenion
Ali, 2013[6]	>4 weeks	TR (>30 mmHg), flat and left-deviated IVS, RV hypertrophy or dilation, steep PA flow curve (AT/ET ratio < 0.3)
An, 2010 [7]	>2 months	TR (≥3 m/s in the absence of PS), flat or left-deviated IVS, RV hypertrophy and dilation
Arattu, 2021[8]	>28 days	TR (>3 m/s)<br>In the absence of TR: flattened or left deviated IVS, right to left shunting across a PFO, ASD, VSD or PDA, RV hypertrophy or RV dysfunction.
Aswani, 2016 [9]	>4 weeks	TR (>3 m/s, RVSP/SBP ratio >0.5), IVS flattening.
Bhat, 2012 [10]	4-6 weeks	TR (>? in the absence of PS), RV hypertrophy, IVS flattening,
Blanca, 2018 [11]	6 months	TR (≥2.8 m/s in the absence of PS), flat or left-deviated IVS
Bruno, 2015 [12]	>36 weeks PMA	TR (> 25 mmHg), RV hypertrophy, IVS flattening
Cartón, 2016 [13]	>2 months	TR (>2,9 m/s)
Check, 2013 [14]	>36 weeks PMA	TR (RVSP/SBP ratio >0.33)<br>Without TR at least two of the following: RV enlargement, RV hypertrophy, IVS flattening and/or abnormal PA Doppler (sawtooth pattern or shortened AT).
Choi, 2015 [15]	>2 months	TR (≥ 3 m/s in the absence of PS)<br>or flat or left-deviated IVS and RV hypertrophy with chamber dilation
Dasgupta, 2018 [16]	36 weeks PMA	TR (>25 mmHg),<br>IVS flattening and/or RV hypertrophy.
DeVries, 2017 [17]	>28 days	TR (>40 mmHg or RVSP/SBP ratio >0.5)<br>Any cardiac shunt with bidirectional or right-to-left flow, or IVS flattening
Gentle, 2023 [18]	>28 days	TR (≥ 35 mmHg), bidirectional flow through the PFO or PDA, or IVS flattening (EI >1.0)
Kanaan, 2018 [19]	>28 days	TR (>3 m/s or >36 mm Hg),<br>elevated PI end-diastolic velocity (>1.5 m/s or >9 mm Hg), right to left shunting, RV dilation, RV hypertrophy, RV dysfunction
Kawai, 2022 [20]	>36 weeks PMA	TR (>?), IVS flattening, AT/ET ratio of PA flow, RV wall thickness and PH score.
Khemani, 2007 [21]	>2 months	TR (>?), RV hypertrophy, IVS flattening or leftward deviation.
Kim, 2014 [38]	unknown	TR (≥ 3 m/s in the absence of PS), or flat or left-deviated IVS and RV hypertrophy and dilation

© 2023 Villamor E et al. *JAMA Network Open.*

Study	Age at echocardiography	Criteria for echocardiographic assessment of pulmonay hypertenion
Kunjunju, 2017 [23]	36 weeks PMA	TR (≥ 2.8 m/s), IVS flattening or leftward deviation (EI >0.81), right-to left PDA shunting >30% of cardiac cycle, TPV/RVETc <0.31, TR/VTI ≥ 0.14.
Lagatta, 2018 [24]	≥34 weeks PMA	Undefined
Lodha, 2022 [25]	36 weeks PMA	TR (>?), IVS flattening, main PA dilation.
Madden 2022 [26]	36 weeks PMA	TR (RVSP/SBP ratio >0.5), right-to-left or bidirectional shunting at any level (ASD, VSD, or PDA) or IVS flattening.
Mourani, 2015 [27]	36 weeks PMA	TR (> 40 mmHg or RVSP/SBP ratio > 0.5), IVS flattening, or any cardiac shunt with bidirectional or right-to-left flow.
Nawaytou, 2022[28]	after 36 weeks PMA	TR (>2.9 m/s), PDA systolic flow velocity (>35 mmHg), IVS flattening (EI>1.0)
Philip 2021[29]	>4 weeks	Cardiac catheterization: PVRi ≥ 3WU*m²
Ra, 2013 [30]	>1 month	TR (≥ 3 m/s in the absence of PS), or flat or left-deviated IVS and RV hypertrophy and dilation
Sallmon, 2022[31]	>3 months	TR (> 2.5 m/s) in the absence of RVOT obstruction
Sheth, 2020 [32]	36 weeks PMA	TR (>40 mmHg or RVSP/SBP ratio >0.5); any VSD or PDA with bidirectional or right-to-left shunting. If no TR or shunt two out of following three criteria: IVS flattening, RV dilation and/or RV hypertrophy.
Slaughter, 2011[33]	> 30 days	TR (RVSP/SBP ratio > 0.5), IVS flattening, RV hypertrophy and/or right to left shunt.
Trittmann, 2014[34]	>28 days	TR (>?) in the absence of PIS, IVS flattening, RV hypertrophy
Vyas-Read, 2017[35]	>30 days	TR (> 32 mmHg), IVS flattening, RV hypertrophy, RV dilation, PDA with bidirectional or right-to-left shunting
Wang, 2022 [36]	> 36 weeks PMA	TR (RVSP/SBP ratio > 0.5), IVS flattened or left-deviated, bidirectional or right-to-left shunt at the PFO or PDA.
Weismann, 2017 [37]	36-38 weeks PMA	TR (>36 mmHg) or IVS flattening.

The numbers in parentheses correspond to the thresholds used to define PH in the different studies. In the case of TR, the value of the jet velocity (m/s) or the estimated RVSP value (mmHg) based on this velocity is given.

ASD = atrial septal defect; AT = acceleration time; EI = eccentricity index; ET = ejection time; IVS = interventricular septum; LV = left ventricular; PA = pulmonary artery; PAP = pulmonary artery pressure; PDA = patent ductus arteriosus; PFO = patent foramen ovale; PH = pulmonary hypertension; PI = pulmonary insufficiency; PMA = postmenstrual age; PR = pulmonary regurgitation; PS = pulmonary stenosis; PVR = pulmonary vascular resistance; RV = right ventricle; RVETc = right ventricular ejection time; RVOT = right ventricular outflow tract; RVSP = right ventricular systolic pressure; SBP = systemic blood pressure; TPV = time to peak velocity; TR = tricuspid valve regurgitation; VSD = ventricular septal defect; VTI = velocity time integral.

© 2023 Villamor E et al. *JAMA Network Open.*

eTable 3. Data on Heterogeneity of the Bayesian Model-Averaged Meta-Analysis (BMA)

Meta-analysis	K	Heterogeneity (Tau)	Standard deviation	95% credible Interval		BF_{rf}	Evidence for		P-value Heterogeneity Frequentist Analysis
				Lower Limit	Upper Limit		Random effects	Fixed effect	
Any PDA	10	0.399	0.181	0.144	0.847	2.20	weak		0.090
Hemodinamically significant PDA	3	0.631	0.427	0.183	1.666	6.03	moderate		0.012
Medically treated PDA	6	0.541	0.300	0.161	1.284	2.19	weak		0.034
Surgically ligated or catheter occluded PDA	16	0.587	0.158	0.349	0.958	$>10^6$	extreme		<0.0001
Medically treated and/or surgically ligated/catheter occluded PDA	8	0.395	0.202	0.131	0.888	0.82		weak	0.249
Prolonged PDA	6	1.373	0.594	0.550	2.826	1872.5	extreme		<0.0001
Time of exposure to PDA	3	0.241	0.178	0.070	0.723	0.54		weak	0.382

BF: Bayes factor; K: number of studies; PDA: patent ductus arteriosus.

© 2023 Villamor E et al. *JAMA Network Open.*

eTable 4. Analysis of Publication Bias by Robust Bayesian Meta-Analysis (RoBMA)

Meta-analysis	K	BF_{10}	BF_{rf}	BF_{bias}	Evidence bias
Any PDA	10	2.05	2.16	0.58	weak/undecided against
Hemodinamically significant PDA	3	1.76	4.98	1.68	weak/undecided for
Medically treated PDA	6	0.42	1.26	1.47	weak/undecided for
Surgically ligated or catheter occluded PDA	16	23.6	$>10^6$	0.46	weak/undecided against
Medically treated and/or surgically ligated/catheter occluded PDA	8	0.69	0.71	0.61	weak/undecided against
Prolonged PDA	6	2.80	794.4	2.29	weak/undecided for
Time of exposure to PDA	3	27.2	0.54	1.37	weak/undecided for

BF: Bayes factor; K: number of studies; PDA: patent ductus arteriosus.

eTable 5. Adjusted Effect Sizes

Study	PDA group	Unadjusted OR (95% CI)	Adjusted OR (95% CI)	Adjustment covariates
Sheth, 2020 [32]	HsPDA	1.18 (0.65-2.14)	0.92 (0.79-1.72)	BW, sex
	SurgPDA	2.20 (1.09-4.46)	1.90 (0.91-3.95)	
Gentle, 2023 [18]	Any PDA	2.94 (1.62-5.35)	4.29 (1.89-9.77)	BW, GA, white race, sex, invasive respiratory support at postnatal day 28, FiO_2 at postnatal day 28
	HsPDA	4.01 (2.08-7.73)	4.15 (1.78-9.64)	

AnyPDA: any ductal shunt detected by echocardiography; BW: birth weight; CI: confidence interval; GA: gestational age; HsPDA: hemodynamically significant PDA; OR: odds ratio; PDA: patent ductus arteriosus; SurgPDA: surgically-ligated or catheter-occludded PDA.

© 2023 Villamor E et al. *JAMA Network Open.*

eReferences

1. Bramer W, Bain P. Updating search strategies for systematic reviews using EndNote. *JMLA.* 2017;105(3):285.
2. Maier M, Bartoš F, Wagenmakers E-J. Robust Bayesian meta-analysis: Addressing publication bias with model-averaging. *Psychol Methods.* 2023;28(1):107.
3. Bartoš F, Maier M, Wagenmakers EJ, Doucouliagos H, Stanley T. Robust Bayesian meta analysis: Model-averaging across complementary publication bias adjustment methods. *Res Synth Methods.* 2023;14(1):99-116.
4. Vevea JL, Hedges LV. A general linear model for estimating effect size in the presence of publication bias. *Psychometrika.* 1995;60:419-435.
5. Stanley TD, Doucouliagos H. Meta-regression approximations to reduce publication selection bias. *Res Synth Methods.* 2014;5(1):60-78.
6. Ali Z, Schmidt P, Dodd J, Jeppesen DL. Predictors of bronchopulmonary dysplasia and pulmonary hypertension in newborn children. *Dan Med J*. Aug 2013;60(8):A4688.
7. An HS, Bae EJ, Kim GB, et al. Pulmonary hypertension in preterm infants with bronchopulmonary dysplasia. *Korean Circul J*. 2010;40(3):131-136.
8. Arattu Thodika FMS, Nanjundappa M, Dassios T, Bell A, Greenough A. Pulmonary hypertension in infants with bronchopulmonary dysplasia: risk factors, mortality and duration of hospitalisation. *J Perinat Med*. Mar 28 2022;50(3):327-333. doi:10.1515/jpm-2021-0366
9. Aswani R, Hayman L, Nichols G, et al. Oxygen requirement as a screening tool for the detection of late pulmonary hypertension in extremely low birth weight infants. *Cardiol Young*. 2016;26(3):521.
10. Bhat R, Salas AA, Foster C, Carlo WA, Ambalavanan N. Prospective analysis of pulmonary hypertension in extremely low birth weight infants. *Pediatrics*. 2012;129(3):e682-e689.
11. Blanca AJ, Duijts L, van Mastrigt E, et al. Right ventricular function in infants with bronchopulmonary dysplasia and pulmonary hypertension: a pilot study. *Pulm Circul*. 2018;9(1):2045894018816063.
12. Bruno CJ, Meerkov M, Capone C, et al. CRIB scores as a tool for assessing risk for the development of pulmonary hypertension in extremely preterm infants with bronchopulmonary dysplasia. *Am J perinatol.* 2015;32(11):1031-1037.
13. Cartón Sánchez AJ. Hipertensión pulmonar estimada por ecocardiografía en prematuros con displasia broncopulmonar: frecuencia, evolución y factores de riesgo. 2016; https://repositorio.uam.es/handle/10486/674863. Accessed January 30, 2023.
14. Check J, Gotteiner N, Liu X, et al. Fetal growth restriction and pulmonary hypertension in premature infants with bronchopulmonary dysplasia. *J perinatol*. 2013;33(7):553-557.
15. Choi EK, Jung YH, Kim H-S, et al. The impact of atrial left-to-right shunt on pulmonary hypertension in preterm infants with moderate or severe bronchopulmonary dysplasia. *Pediatr & Neonatol*. 2015;56(5):317-323.
16. Dasgupta S, Aly AM, Malloy MH, Okorodudu AO, Jain SK. NTproBNP as a surrogate biomarker for early screening of pulmonary hypertension in preterm infants with bronchopulmonary dysplasia. *J Perinatol.* 2018;38(9):1252-1257.
17. DeVries L, Heyne R, Ramaciotti C, et al. Mortality among infants with evolving bronchopulmonary dysplasia increases with major surgery and with pulmonary hypertension. *J Perinatol*. 2017;37(9):1043-1046.
18. Gentle SJ, Travers CP, Clark M, Carlo WA, Ambalavanan N. Patent Ductus Arteriosus and Development of Bronchopulmonary Dysplasia with Pulmonary Hypertension. *Am J Respir Crit Care Med*. 2023;207:921-928
19. Kanaan U, Srivatsa B, Huckaby J, Kelleman M. Association of unit-wide oxygen saturation target on incidence of pulmonary hypertension in very low birthweight premature infants. *J perinatol*. 2018;38(2):148-153.
20. Kawai Y, Hayakawa M, Tanaka T, et al. Pulmonary hypertension with bronchopulmonary dysplasia: Aichi cohort study. *Pediatr Int*. 2022;64(1):e15271.
21. Khemani E, McElhinney DB, Rhein L, et al. Pulmonary artery hypertension in formerly premature infants with bronchopulmonary dysplasia: clinical features and outcomes in the surfactant era. *Pediatrics*. 2007;120(6):1260-1269.
22. Kim DH, Kim HS, Choi CW,et al. Risk factors for pulmonary artery hypertension in preterm infants with moderate or severe bronchopulmonary dysplasia. *Neonatology*.2012; 101:40-46.
23. Kunjunju A, Gopagondanahalli K, Chan Y, Sehgal A. Bronchopulmonary dysplasia-associated pulmonary hypertension: clues from placental pathology. *J Perinatol*. 2017;37(12):1310-1314.
24. Lagatta JM, Hysinger EB, Zaniletti I, et al. The impact of pulmonary hypertension in preterm infants with severe bronchopulmonary dysplasia through 1 year. *J Pediatr*. 2018;203:218-224. e3.

© 2023 Villamor E et al. *JAMA Network Open.*

25. Lodha A, Thomas S, Jain S, et al. Neurodevelopmental Outcomes of Preterm Infants Born< 29 weeks with Bronchopulmonary Dysplasia Associated Pulmonary Hypertension: A Multicenter Study. 2022; Preprint. https://doi.org/10.21203/rs.3.rs-1956482/v1
26. Madden B, Conaway M, Zanelli S, McCulloch M. Screening echocardiography identifies risk factors for pulmonary hypertension at discharge in premature infants with bronchopulmonary dysplasia. *Pediatr Cardiol*. 2022;43(8):1743-1751.
27. Mourani PM, Sontag MK, Younoszai A, et al. Early pulmonary vascular disease in preterm infants at risk for bronchopulmonary dysplasia. *Am J Respir Crit Care Med*. 2015;191(1):87-95.
28. Nawaytou H, Hills NK, Clyman RI. Patent ductus arteriosus and the risk of bronchopulmonary dysplasia-associated pulmonary hypertension. *Pediatr Res*. 2023:1-8.
29. Philip R, Waller BR, Chilakala S, et al. Hemodynamic and clinical consequences of early versus delayed closure of patent ductus arteriosus in extremely low birth weight infants. *J Perinatol*. Jan 2021;41(1):100-108. doi:10.1038/s41372-020-00772-2
30. Ra JJ, Lee SM, Eun HS, et al. Risk factors of pulmonary hypertension in preterm infants with chronic lung disease. Neonat Med. 2013; 20(1):75-80
31. Sallmon H, Koestenberger M, Avian A, et al. Extremely premature infants born at 23-25 weeks gestation are at substantial risk for pulmonary hypertension. *J Perinatol*. 2022;42(6):781-787. doi:10.1038/s41372-022-01374-w
32. Sheth S, Goto L, Bhandari V, Abraham B, Mowes A. Factors associated with development of early and late pulmonary hypertension in preterm infants with bronchopulmonary dysplasia. *J Perinatol*. 2020;40(1):138-148.
33. Slaughter JL, Pakrashi T, Jones DE, South AP, Shah TA. Echocardiographic detection of pulmonary hypertension in extremely low birth weight infants with bronchopulmonary dysplasia requiring prolonged positive pressure ventilation. *J Perinatol*. 2011;31(10):635-40. doi:10.1038/jp.2010.213
34. Trittmann JK, Nelin LD, Zmuda EJ, et al. Arginase I gene single-nucleotide polymorphism is associated with decreased risk of pulmonary hypertension in bronchopulmonary dysplasia. *Acta Paediatr*. 2014;103(10):e439-43. doi:10.1111/apa.12717
35. Vyas-Read S, Kanaan U, Shankar P, et al. Early characteristics of infants with pulmonary hypertension in a referral neonatal intensive care unit. *BMC Pediatr*. 2017;17(1):163. doi:10.1186/s12887-017-0910-0
36. Wang C, Ma X, Xu Y, Chen Z, Shi L, Du L. A prediction model of pulmonary hypertension in preterm infants with bronchopulmonary dysplasia. *Front Pediatr*. 2022;10:925312.
37. Weismann C, Asnes J, Bazzy-Asaad A, Tolomeo C, Ehrenkranz R, Bizzarro M. Pulmonary hypertension in preterm infants: results of a prospective screening program. *J Perinatol*. 2017;37(5):572-577.

© 2023 Villamor E et al. *JAMA Network Open.*
