# Supplementary material for: Patent Ductus Arteriosus and Bronchopulmonary Dysplasia–Associated Pulmonary Hypertension: A Bayesian Meta-Analysis
Source: JAMA Netw Open. 2023 Nov 28;6(11):e2345299. doi: 10.1001/jamanetworkopen.2023.45299 (PMC10685885; doi:10.1001/jamanetworkopen.2023.45299)
Supplement: Supplement 2. — Data Sharing Statement [file jamanetwopen-e2345299-s002.pdf]

Data Sharing Statement

Villamor. Patent Ductus Arteriosus and Bronchopulmonary Dysplasia–Associated Pulmonary Hypertension. *JAMA Netw Open*. Published November 28, 2023.
doi:10.1001/jamanetworkopen.2023.45299

Data
Data available: No

Additional Information
Explanation for why data not available: The study is a meta-analysis based on published data
